# Sex-Specific Differences in Gut Microbiota Composition in Adult Patients with Bronchial Asthma

**DOI:** 10.3390/biomedicines14010125

**Published:** 2026-01-08

**Authors:** Chihiro Hirano, Yutaka Kozu, Yusuke Jinno, Yusuke Kurosawa, Shiho Yamada, Kouta Hatayama, Kanako Kono, Kenji Mizumura, Motoyasu Iikura, Shuichiro Maruoka, Hiroaki Masuyama, Yasuhiro Gon

**Affiliations:** 1 Symbiosis Solutions Inc., Tokyo 101-0064, Japan; hirano@symbiosis-solutions.co.jp (C.H.); hatayama@symbiosis-solutions.co.jp (K.H.); kono@symbiosis-solutions.co.jp (K.K.); masuyama@symbiosis-solutions.co.jp (H.M.); 2 Division of Respiratory Medicine, Department of Internal Medicine, Nihon University School of Medicine, Tokyo 173-8610, Japan; kozu.yutaka@nihon-u.ac.jp (Y.K.); jinno.yusuke@nihon-u.ac.jp (Y.J.); kurosawa.yusuke@nihon-u.ac.jp (Y.K.); yamada.shiho22@nihon-u.ac.jp (S.Y.); mizumura.kenji@nihon-u.ac.jp (K.M.); iikura.motoyasu@nihon-u.ac.jp (M.I.); gon.yasuhiro@nihon-u.ac.jp (Y.G.)

**Keywords:** bronchial asthma, dysbiosis, gut microbiota, sex differences, short-chain fatty acids

## Abstract

**Background:** Gut microbiota dysbiosis has been associated with childhood asthma; however, its role in adult bronchial asthma (BA), particularly in Japanese populations, remains unclear. The potential influence of sex-based differences also warrants investigation. We aimed to investigate the association between gut microbiota composition and adult BA in a Japanese cohort, focusing on sex-specific differences. **Methods:** Stool samples from 108 Japanese adults with BA (48 male and 60 female individuals) and 210 healthy controls (90 male and 120 female individuals) were analyzed using 16S rRNA gene sequencing. Analyses were stratified by sex. β-diversity was assessed using non-metric multidimensional scaling and permutational multivariate analysis of variance. Genus-level taxonomic comparisons were conducted using the ANOVA-Like Differential Expression version 2 tool on centered log-ratio-transformed data. **Results:** β-diversity significantly differed between the groups among both male and female individuals. In male individuals, 11 taxa had absolute effect sizes of ≥0.2. In female individuals, 19 taxa met this threshold, with 8 reaching significance after Benjamini–Hochberg correction. *Streptococcus* and *Blautia* were enriched in the BA group in both sexes, whereas other taxa showed sex-specific patterns, such as *Veillonella* in male and *Flavonifractor* and *Eggerthella* in female individuals. Several short-chain fatty acid (SCFA)-producing taxa were depleted in the BA group. **Conclusions:** Our findings suggest that gut microbiota dysbiosis occurs in Japanese adults with BA, characterized by enrichment of taxa associated with respiratory diseases and depletion of SCFA-producing bacteria. The observed patterns highlight the importance of considering sex-specific differences in future research.

## 1. Introduction

In recent years, gut microbiota dysbiosis has been associated with the onset of childhood bronchial asthma (BA) [1,2,3]. In particular, the gut microbiota during infancy influences the risk of developing BA later in childhood [4,5]. In childhood BA, abnormal production of microbial metabolites affects immune response [3]. Short-chain fatty acids (SCFAs) such as butyric, propionic, and acetic acid—produced by gut bacteria—have a protective effect against airway inflammation. Reduced levels of these SCFAs in stool samples have been associated with an increased risk of childhood asthma [6,7]. The epidemiological background of BA is summarized in the Supplementary Text. Gut microbiota may also be associated with the onset and severity of BA in adults. Obesity and diet-related habits influence both the development and progression of BA by altering gut microbiota composition and its metabolic products [8,9,10,11,12]. A study of British adults found that patients with asthma had higher levels of *Eggerthella lenta*, *Clostridium bolteae* (now validly published as *Enterocloster bolteae*), *Clostridium ramosum* (currently *Thomasclavelia ramosa*, synonym: *Erysipelatoclostridium ramosum*), and *Clostridium spiroforme* (*Thomasclavelia spiroformis*) compared with those found in healthy controls. These patients also showed a reduction in the SCFA-producing bacterium *Faecalibacterium prausnitzii* [12]. Similarly, adults with BA exhibit lower levels of SCFA-producing bacteria, such as *Faecalibacterium* and *Oscillibacter* [12,13,14]. These findings suggest that gut microbiota may influence asthma pathogenesis by modulating T cell activity through the production of SCFAs [12,15]. However, most of these studies were conducted in Europe, the United States, Colombia, and China and did not involve Japanese populations [12,13,14,16,17,18,19]. The composition of the gut microbiota varies by country and region, and Japanese populations have a distinct microbial profile [20,21]. Therefore, findings from studies conducted in other countries may not be directly applicable to the Japanese population. To better understand the relationship between adult BA and the gut microbiota in Japan—and to potentially inform treatment strategies—research specifically involving Japanese populations is required. However, to the best of our knowledge, no such studies have been conducted in Japan. Additionally, although sex differences are well-documented in both the prevalence of adult BA and gut microbiota composition [22,23,24], previous studies have not adequately accounted for these differences. Most analyses combined data from both sexes, potentially obscuring important sex-specific associations. We aimed to investigate whether gut microbiota dysbiosis occurs in Japanese adults with BA, with a specific focus on sex-specific differences [20,21,24,25,26].

## 2. Materials and Methods

### 2.1. Study Design

This study employed a cross-sectional observational design to compare the gut microbiota composition of adult Japanese patients with BA with that of healthy controls. Stool samples were collected at a single time point, and gut microbiota analyses were conducted separately for male and female individuals.

### 2.2. Ethical Considerations

This study was conducted in accordance with the Declaration of Helsinki and approved by the Institutional Review Boards of Nihon University School of Medicine, Itabashi Hospital (Approval No. RK-210608-14; Approval date: 8 June 2021), as well as the Institutional Review Board of Shiba Palace Clinic, Tokyo, Japan (Approval Nos. 144131_rn-27593, Approval date: 9 January 2020; and 145968_rn-29327, 12 November 2020). Written informed consent was obtained from all participants.

### 2.3. Study Population

A total of 108 adult Japanese patients (48 male and 60 female individuals) diagnosed with BA and receiving outpatient care at Nihon University Itabashi Hospital were recruited and classified into the BA group. The age distribution of this group is shown in Table S1. Although the BA group included participants undergoing long-term controller medication and presenting with various comorbid conditions (Table S2), no formal adjustment for these potential confounders was performed, owing to the limited sample size. Stool samples from the BA group were collected between September 2021 and July 2022. For the control group, data were obtained from users of a gut microbiota testing service operated by Symbiosis Solutions Inc. (Tokyo, Japan), collected between April 2020 and March 2023. Individuals who reported not having any of the diseases listed in Table S3 or any other diseases, and who had not taken antibiotics within the previous 3 months were eligible for inclusion. From this healthy population, participants were randomly selected to match the age distribution of the participants in the BA group. The control group included 120 female individuals—twice the number of female participants in the BA group. Regarding male individuals, 90 controls—approximately twice the size of the male participants in the BA group—were selected, except for those in their 70s. In that age range, the number of available healthy male individuals was limited; therefore, all eligible individuals in their 70s were included. The age distribution of the control group is presented in Table S1. Stool samples from the control group were collected between April 2020 and March 2023. To minimize potential impacts on gut microbiota composition, the exclusion criteria for both groups were refusal to participate, current pregnancy or breastfeeding, and recent antibiotic use (within the previous 3 months), as well as insufficient gut microbiota data, non-Japanese ethnicity, or enema-derived stool samples.

### 2.4. Questionnaire Survey

Background information—including age, sex, height, weight, and general health status—was collected via self-administered questionnaires completed at the time of stool sample collection. For participants in the BA group, clinical information, such as disease status, treatment history, and current medications, was obtained through medical examinations.

### 2.5. Collection of Stool Samples and 16S rRNA Gene Sequence Data Analysis

Stool sample collection, DNA extraction, and 16S rRNA gene sequencing (targeting variable regions V1–V3) using the MiSeq platform (Illumina, San Diego, CA, USA) were conducted following the protocol described by Hatayama et al. [24] Amplicon Sequence Variants (ASVs) were generated using the DADA2 v1.16.0 package [27] in the R software v4.0.3 (R Foundation for Statistical Computing, Vienna, Austria) [28]. Taxonomic classification of ASVs was performed using the Ribosomal Database Project training set v18 [29] (available at: https://zenodo.org/record/4310151#.ZDUBAXbP2Ht; accessed on 11 April 2023).

### 2.6. Gut Microbiota Analysis

Microbial diversity and composition were assessed using standard bioinformatics tools [30,31,32]. The α-diversity indices were calculated at the genus level, using vegan 2.6-2 in Rv. 4.2.0. Differential abundance analyses were performed using ANOVA-Like Differential Expression version 2 (ALDEx2) tool. β-diversity was visualized with non-metric multidimensional scaling (NMDS) based on the Bray–Curtis index, and group differences were evaluated using permutational multivariate analysis of variance (PERMANOVA) and permutational analysis of multivariate dispersions (PERMDISP). Full methodological details, including software versions and statistical settings, are provided in the Supplementary Text [6,7].

### 2.7. Statistical Analysis

Group comparisons were performed using the Wilcoxon rank-sum test with the wilcox.test function in R (v4.2.0), with paired = FALSE and correct = FALSE. Statistical significance was set at *p* < 0.05.

## 3. Results

### 3.1. Demographic Characteristics

There were no significant age differences between the BA and control groups for either sex (Table 1). No significant differences in the body mass index (BMI) were observed among male individuals; however, among female individuals, the BMI was significantly higher in the BA group than in the control group (Table 1).

### 3.2. Comparison of Gut Microbiota Between the BA and Control Groups

α-diversity was assessed using Shannon, Simpson, Pielou’s evenness, and Chao1 indices. The Shannon and Simpson indices reflect the richness and evenness, Pielou’s index reflects evenness, and Chao1 reflects richness. A significant difference between the BA and control groups was observed only for Pielou’s evenness index in male individuals (Table 2). Among female individuals, no significant intergroup differences in α-diversity indices were observed (Table 2).

**Figure 1:**
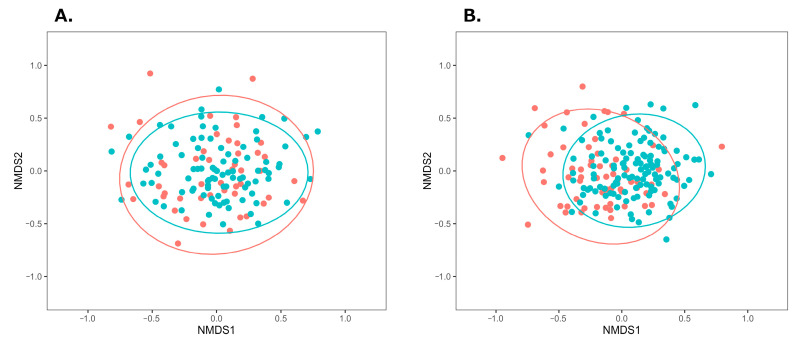
NMDS plots of gut microbiota in the BA and control groups using the Bray–Curtis index. (**A**) Male individuals (stress = 0.219), (**B**) Female individuals (stress = 0.223). Ellipses indicate 95% confidence intervals around the centroids. Red and green plots represent the BA and control groups, respectively. Abbreviations: BA, bronchial asthma; NMDS, non-metric multidimensional scaling.

β-diversity was visualized with NMDS plots based on the Bray-Curtis index. Among male individuals, NMDS plots in the BA group showed a wider distribution, compared with those in the control group (Figure 1A). Tests for homogeneity of multivariate dispersions (PERMDISP) showed no significant differences (*p* = 0.697), whereas PERMANOVA indicated a significant group difference (*p* < 0.001). For female individuals (Figure 1B), the BA group’s distribution was more left-skewed than the control group’s distribution, with significant differences observed in β-diversity (PERMDISP *p* = 0.703, PERMANOVA *p* = 0.003). These results suggest distinct gut microbiota compositions between the BA and control groups in both sexes.

**Figure 2:**
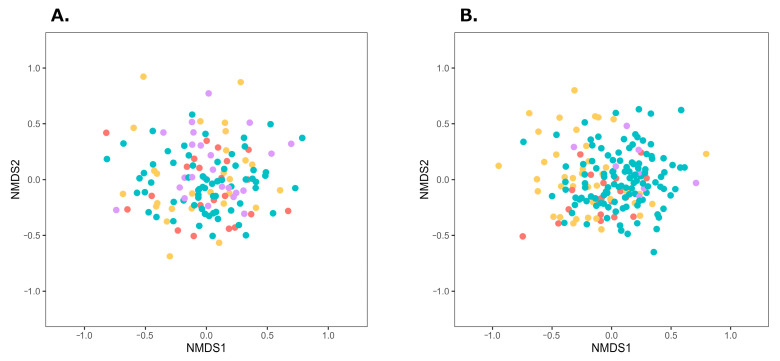
NMDS plots of gut microbiota in the four subgroups divided by obesity status within the BA and control groups. Distances were calculated using the Bray–Curtis index. (**A**) Male individuals (stress = 0.219), (**B**) Female individuals (stress = 0.223). Red, yellow, purple, and green plots represent BA-obese, BA-non-obese, control-obese, and control-non-obese groups, respectively. Abbreviations: BA, bronchial asthma; NMDS, non-metric multidimensional scaling.

Obesity and obesity-inducing dietary habits have been reported to influence the onset and severity of BA through gut microbiota dysbiosis [8,9,10,11,12]. In this study, female patients with BA had significantly higher BMI, compared with the female participants in the control group. To adjust for the effects of obesity, the participants in both groups were divided into obese and non-obese subgroups, and β-diversity of the gut microbiota was compared within each subgroup. According to the Japan Society for the Study of Obesity, individuals with a BMI of 18.5–25 kg/m^2^ are classified as having normal weight, whereas those with a BMI ≥ 25 kg/m^2^ are classified as obese [33]. In this study, following this criterion, individuals with a BMI ≥ 25 kg/m^2^ were classified as obese, and those with a BMI < 25 kg/m^2^ were classified as non-obese. Obese and non-obese individuals in the BA and control groups were further classified by sex and plotted on an NMDS plot (Figure 2). Among male individuals, significant differences were observed between the BA obese and control obese subgroups, as well as between the corresponding non-obese subgroups (male individuals, obese subgroup: PERMDISP *p* = 0.981, PERMANOVA *p* = 0.022; male individuals, non-obese subgroup: PERMDISP *p* = 0.764, PERMANOVA *p* < 0.001). Among female individuals, a significant difference was observed between the non-obese subgroups of the BA and control groups (PERMDISP *p* = 0.832, PERMANOVA *p* = 0.008); however, no significant difference was found between the BA obese and control obese subgroups (PERMDISP *p* = 0.238, PERMANOVA *p* = 0.196). These subgroup analyses indicate that gut microbiota composition differed between the BA and control groups in both obese and non-obese male individuals, whereas in female individuals, the difference was only observed in the non-obese subgroups.

**Figure 3:**
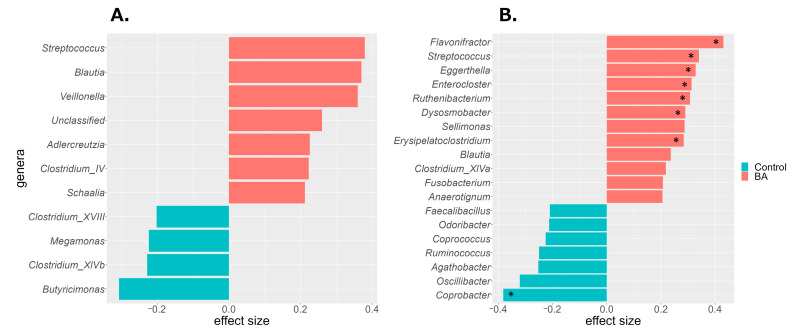
Differentially abundant bacterial taxa identified by ALDEx2 analysis in the BA and control groups. Taxa with an absolute effect size ≥ 0.2 are shown for (**A**) male and (**B**) female individuals. Red indicates enrichment in the BA group; green indicates enrichment in the control group. Asterisks denote significant differences (Wilcoxon rank-sum test with Benjamini–Hochberg correction, * *p* < 0.05). BA, bronchial asthma.

To identify gut bacterial taxa (genus level) that differed between the BA and control groups, ALDEx2 analysis was performed. Differences in centered log ratio (CLR)-transformed bacterial abundance were expressed as effect sizes. Taxa with an effect size > 0.2 were considered more abundant in the BA group, whereas those with an effect size < −0.2 were less abundant. In male patients with BA, *Streptococcus*, *Blautia*, *Veillonella*, Unclassified (a taxon comprising ASVs that could not be classified), *Adlercreutzia*, *Clostridium*_IV, and *Schaalia* were more abundant, whereas *Butyricimonas*, *Clostridium*_XlVb, *Megamonas*, and *Clostridium*_XVIII were less abundant (Figure 3A). In female patients with BA, *Flavonifractor*, *Streptococcus*, *Eggerthella*, *Enterococcus*, *Ruthenibacterium*, *Dysosmobacter*, *Sellimonas*, *Erysipelatoclostridium*, *Blautia*, *Clostridium*_XlVa, *Fusobacterium*, and *Anaerotignum* were more abundant, whereas *Coprobacter*, *Oscillibacter*, *Agathobacter*, *Ruminococcus*, *Coprococcus*, and *Odoribacter* were less abundant (Figure 3B). Among these, eight taxa in female individuals showed significant differences in CLR abundance (*p* < 0.05). Both sexes exhibited a higher prevalence of *Streptococcus* and *Blautia* in the BA group, although other taxa differed by sex.

## 4. Discussion

To the best of our knowledge, this is the first study to investigate the gut microbiota of adult Japanese patients with BA. We found that the gut microbiota composition differed between male and female patients as well as healthy controls, suggesting the presence of gut microbiota dysbiosis in adult Japanese patients with asthma. Previous studies comparing adult patients with asthma and healthy controls have reported significant differences in β-diversity, consistent with our findings [14,17,19]. This indicates that gut microbiota dysbiosis is common among adult patients with asthma across countries and regions. However, results regarding α-diversity have been inconsistent across studies [13,14,16,19,34]. In our study, no significant differences were observed in α-diversity indices between the BA and control groups, except for Pielou’s evenness index in male patients with BA. This suggests that dysbiosis may not always be reflected by changes in α-diversity, making it difficult to distinguish between the two groups based solely on these measures. Obesity is a known risk factor for asthma onset and severity [8,35,36,37,38], and obesity-promoting diets have been reported to affect BA through gut microbiota dysbiosis [8,12,39,40,41,42]. If the gut microbiota differences observed in Figure 1 were solely due to obesity-related dysbiosis, no differences would be expected between the BA and control groups, when comparing the non-obese subgroups. Significant differences were observed between the BA and control groups in both obese and non-obese male individuals, as well as in non-obese female individuals. These results indicate that gut microbiota dysbiosis in BA—except for obese female individuals—is characteristic of the disease itself rather than obesity. However, no significant differences were observed between the obese female individuals in the BA and the control groups. These results suggest that the gut microbiota of obese female patients with BA may be similar to that of the female control participants. Previous studies have shown that obesity-promoting high-fat, low-fiber diets increase inflammatory gut bacteria and decrease SCFA-producing bacteria, influencing the onset and severity of BA and other inflammatory diseases [8,9,10,11,12,39,40,41,42]. Based on this, it can be inferred that obese female individuals in the BA and control groups share similar gut microbiota, potentially linked to obesity and/or related dietary habits. However, owing to the small number of obese female participants in the control group (Table S4) and the absence of a nutritional survey to assess dietary intake in this study, further investigation with larger samples and detailed dietary analysis is required to clarify the relationship between obesity, gut microbiota, and BA.

In this study, gut microbiota dysbiosis in the BA group was characterized by higher abundance of *Streptococcus* and *Blautia* than that in the control group in both sexes. A previous study conducted in Finland also reported positive associations between these taxa and adult BA development [13]. The high relative abundance of *Streptococcus* and *Blautia* suggests that these bacteria characterize gut microbiota dysbiosis in Japanese patients with BA, regardless of sex. *Streptococcus* is a predominant oral bacterium in patients with BA [43,44,45,46,47]. As bacteria from the respiratory tract or oral cavity can enter the digestive tract via saliva, the *Streptococcus* found in the BA group may originate from these sites. Similarly, *Blautia* is a resident bacterium that produces acetic acid, an SCFA; however, its excessive proliferation has been reported to be associated with the worsening of asthma and allergic diseases [13,48]. Notably, reduced immunoglobulin (Ig) A binding to *Blautia* has been observed in childhood asthma, which may reflect a breakdown of immune control mechanisms [49]. Such abnormal IgA responses may promote *Blautia* overgrowth, contributing to intestinal inflammation. It is also important to consider the effects of medications, as many drugs, including antibiotics, can alter gut microbiota composition, particularly affecting *Streptococcus* and *Blautia* abundances [50,51,52]. The BA group in this study included individuals undergoing medical treatment; therefore, it is possible that medication use contributed to the changes in their gut microbiota. An increased abundance of *Veillonella*, characteristic of gut microbiota dysbiosis in male patients with BA, has been associated with higher asthma incidence in adulthood [13]. Additionally, the relative abundance of *Schaalia* in sputum correlates with clinical outcomes in patients with chronic obstructive pulmonary disease (COPD) and may promote inflammatory responses [53]. Several gut taxa enriched in female patients with BA have also been associated with asthma and COPD. For example, patients with asthma exhibit higher abundance of *Eggerthella lenta*, *Clostridium bolteae* (now *Enterocloster*), *Clostridium clostridioforme* (now *Enterocloster clostridioformis*), and *Flavonifractor* [12,13,19,54]. Moreover, *Flavonifractor* and *Ruthenibacterium* have been linked to COPD onset [13,55]. Although many abundant taxa in the BA group differed between male and female individuals, they are commonly associated with diseases such as asthma and COPD that impair respiratory function through bronchial inflammation. *Butyricimonas*, *Clostridium*_XlVb, and *Megamonas*—which were less abundant in male patients with BA—and *Coprobacter*, *Oscillibacter*, *Agathobacter*, *Ruminococcus*, *Coprococcus*, and *Odoribacter*—which were less abundant in female patients with BA— are all gut bacteria that produce SCFAs [56,57,58,59,60,61,62,63,64]. Although the specific taxa differed between the sexes, it is notable that SCFA-producing bacteria were consistently less abundant in the BA group versus the control group. Similar to our findings, previous studies have reported reduced SCFA-producing bacteria and lower SCFA levels in the stool collected from patients with asthma [12,65]. SCFAs regulate immune cell functions by activating their receptors and influencing gene expression, differentiation, chemotaxis, proliferation, and apoptosis, thereby playing a key role in inflammation control [10,15,66,67,68,69,70]. Moreover, SCFAs are considered critical immunomodulatory agents in the gut–lung axis—a crosstalk between the gut microbiota and lungs, essential for immune homeostasis [10,71]. Altered gut microbiota composition has been linked to lung diseases, such as asthma, COPD, cystic fibrosis, and tuberculosis [72]. A reduction in SCFA-producing bacteria may decrease circulating SCFA levels, potentially contributing to increased airway inflammation. *Agathobacter*, which was less abundant in female patients with BA, and *Butyricimonas*, which tended to be less abundant in male patients with BA, are known hydrogen producers [60,61,73]. Hydrogen is small enough to diffuse into the body and is recognized for its anti-inflammatory properties [73]. A reduction in hydrogen production may contribute to chronic inflammation in BA. Additionally, lower intestinal hydrogen concentrations may promote the oxidation of bile acids by *Eggerthella* lenta, leading to a shift in the hydrophobic–hydrophilic balance of the bile acid pool in the colon [74,75,76]. This shift can alter gut microbiota structure and promote dysbiosis. Co-culture experiments have shown that bile acid oxidation by *Eggerthella lenta* reduces bile acid toxicity and creates favorable conditions for the growth of *Erysipelatoclostridium ramosum* [77]. In the present study, *Eggerthella* and *Erysipelatoclostridium* were more abundant in female patients with BA, and it is possible that certain phenomena promoting this dysbiosis occurred. Notably, the prevalence of adult BA is higher in female than in male individuals [22,23], which may be related to a female-specific dysbiosis of the gut microbiota. The taxonomic profiles of gut bacteria associated with dysbiosis in the Japanese BA group differed between male and female individuals, except for *Streptococcus* and *Blautia*, which were common to both. This may reflect inherent sex differences in the gut microbiota of Japanese individuals [24]. Such differences are thought to result from complex interactions between biological and environmental factors, with sex hormones, such as estrogen and androgen, known to influence gut microbiota composition [78,79]. In this study, we analyzed male and female individuals separately and revealed that many dysbiosis-related taxa differed based on sex. However, in mixed-sex analyses, these effects may be masked by sex-based differences and thus overlooked. Indeed, in the combined analysis of all participants (108 in the BA group and 210 in the control group), taxa more abundant in the BA group (ALDEx2, effect size ≥ 0.2) included *Streptococcus*, *Blautia*, *Flavonifractor*, *Veillonella*, *Eggerthella*, *Enterocloster*, and *Sellimonas* (Figure S1). Taxa less abundant in the BA group (effect size ≤ −0.2) were *Coprobacter* and *Butyricimonas*. These taxa overlapped with those identified in the sex-stratified analyses, underscoring the importance of considering sex differences when studying the gut microbiota in BA. Additionally, depending on the sex ratio of the study population, results may be biased toward one sex. Most studies investigating the association between adult BA and the gut microbiota have not accounted for sex differences [12,13,14,16,17,19]. The low consistency in the taxonomic groups of gut bacteria associated with BA across multiple studies may reflect regional and national differences in gut microbiota composition. However, variations in sex composition among study populations may also have contributed. To minimize such confounding, it is advisable to analyze gut microbiota data separately by sex. In the present study, *Adlercreutzia* and *Clostridium*_IV were more abundant in male patients with BA than in male control participants, whereas *Ruthenibacterium*, *Dysosmobacter*, *Sellimonas*, *Clostridium*_XlVa, *Fusobacterium*, and *Anaerotignum* were more abundant in female patients with BA than in female control participants. Currently, no reports suggest an association between these taxa and BA. Although these bacteria have not previously been identified as characteristics of BA, future studies may reveal clear associations. The findings of this study indicate that gut microbiota dysbiosis occurs in Japanese adults with BA. This suggests that the gut microbiota may be a potential target for improving or preventing adult BA, which is difficult to treat. In the future, interventions such as dietary modifications, prebiotics, and probiotics may offer new strategies for managing BA. Furthermore, tailoring such interventions according to sex and individual gut microbiota profiles may lead to more favorable outcomes.

### Limitations of the Study

This study has some limitations, which are outlined below.

1. This was a pilot study with a relatively small sample size. To further elucidate the changes in gut microbiota observed in patients with asthma in the present study, future research with larger sample sizes is necessary.
2. Most individuals in the BA group had already started treatment, which may have influenced their gut microbiota. Many asthma medications are known to alter the airway and gut microbiota composition [80,81]. Therefore, it is difficult to determine whether the dysbiosis of the gut microbiota observed in this study is due to the disease itself or the effects of treatment.
3. Some participants had comorbidities, potentially confounding the results.
4. The study population was limited to the Japanese population, which restricts the generalizability of the findings to other ethnic or geographic populations.
5. The stool samples were collected at a single time point. Individuals who had taken antibiotics within the previous 3 months were excluded; however, the gut microbiota composition may be influenced by multiple factors, such as recent diet and environment.

The possibility that the gut microbiota characteristics observed in the BA group were affected by treatment or other underlying conditions cannot be ruled out. Although this study confirmed the presence of gut microbiota dysbiosis in Japanese patients with BA, the findings do not determine causality, nor necessarily identify the gut bacteria involved in BA onset. To clarify the causality between changes in the gut microbiota and the onset of asthma, longitudinal studies are necessary. Furthermore, this study demonstrated that sex is an important factor to be considered; however, there are various other factors that influence the gut microbiota, such as dietary habits, living environment, and pet ownership.

Future research should involve comparative analyses of the gut microbiota before and after therapeutic intervention, ideally after controlling for potential confounding factors. Considering the influence of ethnicity and geographic factors on gut microbiota composition, future studies are warranted to evaluate the relevance of these findings in non-Japanese cohorts.

## 5. Conclusions

In this study, we compared the gut microbiota of adult Japanese patients with BA and healthy controls by sex, revealing the presence and characteristics of gut microbiota dysbiosis in the BA group. Many taxa enriched in the BA group have previously been associated with respiratory diseases, whereas those depleted were often SCFAs-producing bacteria. Notably, the gut bacterial taxa associated with BA differed between male and female individuals. These findings suggest that gut microbiota dysbiosis represents a novel target for the prevention and management of adult BA.

## Figures and Tables

**Table 1:** Characteristics of participants in the BA and control groups.

		Male Individuals			Female Individuals	
	BA Group (*n* = 48)	Control Group (*n* = 90)	*p*-Value *	BA Group (*n* = 60)	Control Group (*n* = 120)	*p*-Value *
Age (years)	59.1 ± 13.1	56.7 ± 13.0	0.247	52.1 ± 15.7	51.0 ± 16.2	0.636
BMI (kg/m^2^)	24.5 ± 4.4	23.3 ± 2.8	0.129	24.3 ± 5.5	20.8 ± 2.4	**<0.001**

Values are presented as mean ± standard deviation. Abbreviations: BA, bronchial asthma; BMI, body mass index. * *p*-values were calculated using the Wilcoxon rank-sum test. Bolded *p*-values indicate significant differences between the BA and control groups.

**Table 2:** α-diversity indices of gut microbiota in the BA and control groups.

α-Diversity Indices	Male Individuals			Female Individuals		
	BA Group (*n* = 48)	Control Group (*n* = 90)	*p*-Value *	BA Group (*n* = 60)	Control Group (*n* = 120)	*p*-Value *
Shannon	2.60 ± 0.32	2.64 ± 0.30	0.418	2.61 ± 0.28	2.62 ± 0.24	0.851
Simpson	0.87 ± 0.05	0.88 ± 0.04	0.291	0.88 ± 0.04	0.88 ± 0.03	0.985
Pielou’s evenness	0.67 ± 0.07	0.70 ± 0.06	**0.024**	0.68 ± 0.05	0.69 ± 0.05	0.404
Chao1	53.90 ± 17.94	47.95 ± 14.67	0.076	51.05 ± 12.34	49.66 ± 13.95	0.378

Values are presented as mean ± standard deviation. Abbreviations: BA, bronchial asthma. * *p*-values were calculated using the Wilcoxon rank-sum test. Bolded *p*-values indicate significant differences between the BA and control groups.

## Data Availability

The data presented in this study are available on request from the corresponding author due to privacy and confidentiality concerns.

## Supplementary Materials

The following supporting information can be downloaded at https://www.mdpi.com/article/10.3390/biomedicines14010125/s1, Figure S1: Characteristic gut bacterial taxa in the mixed-sex BA group identified using the ALDEx2 analysis; Table S1: Age distribution of participants in the BA and control groups; Table S2: Comorbid diseases in participants with BA; Table S3: List of diagnosed diseases confirmed at the time of stool sample collection for the gut microbiota testing service; Table S4: Distribution of participants by obesity status in the BA and control groups, stratified by sex.

## Abbreviations

The following abbreviations are used in this manuscript:

ALDEx2	ANOVA-Like Differential Expression version 2
ASVs	Amplicon Sequence Variants
BA	Bronchial asthma
BMI	Body mass index
COPD	Chronic obstructive pulmonary disease
Ig	Immunoglobulin
NMDS	Non-metric multidimensional scaling
PERMANOVA	Permutational multivariate analysis of variance
PERMDISP	Permutational analysis of multivariate dispersions
SCFA	Short-chain fatty acid
