# Supplementary material for: Sex-Specific Differences in Gut Microbiota Composition in Adult Patients with Bronchial Asthma
Source: Biomedicines. 2026 Jan 8;14(1):125. doi: 10.3390/biomedicines14010125 (PMC12838268; doi:10.3390/biomedicines14010125)
Supplement: Supplementary file 1 [file biomedicines-14-00125-s001.zip › biomedicines-3994161-supplementary.pdf]

Supplementary Materials

SUPPLEMENTARY TEXT

Epidemiological Background

Bronchial asthma (BA) is a chronic respiratory disease affecting approximately 300 million people worldwide. Contraction of the bronchial smooth muscles leads to airway narrowing and restricted airflow, resulting in symptoms such as wheezing, coughing, and difficulty breathing [25]. These symptoms impose both physical and psychological burdens on patients and significantly reduce their quality of life [26]. In Japan, the prevalence of BA is estimated to be approximately 7% in children and 4% in adults. According to the Ministry of Health, Labor, and Welfare's 2020 patient survey, the estimated number of patients with BA is approximately 918,000. Among adults aged 20 years and older, over 60% of BA cases begin in adulthood. Among patients aged 40 years or older, adult-onset asthma accounts for more than 70% of cases [22]. Globally, adult BA is more prevalent in female than in male individuals [22,23]. (Source: Most Recent National Asthma Data 2021, https://www.cdc.gov/asthma/most_recent_national_asthma_data.htm).

Gut Microbiota Analysis

The α-diversity indices (Shannon, Simpson, Pielou's evenness, Chao1) were calculated at the genus level, using vegan 2.6-2 in Rv. 4.2.0, and inter-group comparisons of gut microbiota were performed using the ANOVA-Like Differential Expression version 2 (ALDEx2) tool, as described by Hatayama et al. Microbial abundance count data were transformed into centered log-ratio values. For group comparisons, the Wilcoxon rank-sum test was used, with multiple testing correction applied using the Benjamini–Hochberg method via the *p*.adjust function (method = "BH").

For visualization of β-diversity, non-metric multidimensional scaling (NMDS) based on the Bray–Curtis index was performed using the metaMDS function from the vegan package (v2.6-4) in R (v4.2.0). Permutational multivariate analysis of variance was conducted using the adonis function in vegan (permutations = 9999) to test for group differences. Additionally, permutational multivariate analysis of dispersion, which assesses multivariate homogeneity of group dispersions, was performed using the betadisper function from the same package [32].

SUPPLEMENTARY FIGURE

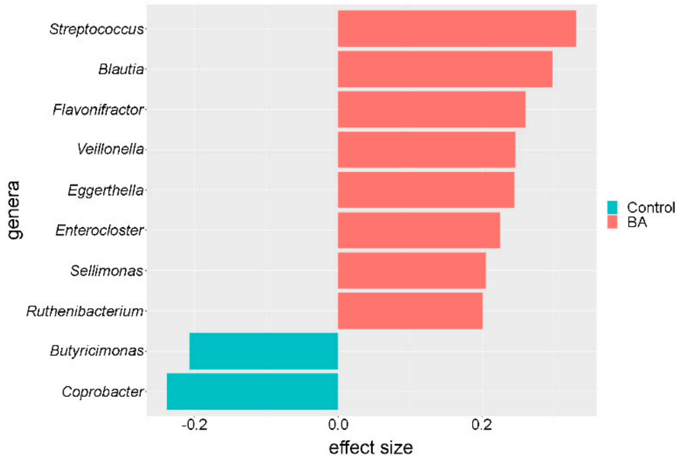

Figure S1. Characteristic gut bacterial taxa in the mixed-sex BA group identified using the ALDEx2 analysis. Taxa with an absolute effect size ≥ 0.2 are shown. Red bars indicate enrichment in the BA group, and green bars indicate enrichment in the controls. Asterisks denote statistically significant differences (Wilcoxon rank-sum test, Benjamini–Hochberg correction,* $p < 0.05$). BA, bronchial asthma

SUPPLEMENTARY TABLES

Table S1. Age distribution of participants in the BA and control groups

Age group (years)	Male individuals		Female individuals	
	BA group (*n* = 48)	Control group (*n* = 90)	BA group (*n* = 60)	Control group (*n* = 120)
20s (20–29)	2	4	7	14
30s (30–39)	3	6	9	18
40s (40–49)	6	12	12	24
50s (50–59)	10	20	10	20
60s (60–69)	15	30	11	22
70s (70–79)	12	18	11	22

Abbreviations: BA, bronchial asthma

Table S2. Comorbid diseases in patients with BA

Disease	Male patients	Female patients
Infection		
Herpes zoster	1	0
Tumors		
Breast cancer	0	1
Cervical cancer	0	1
Prostate cancer	1	0
Uterine leiomyoma	0	2
Thyroid tumor	0	1
Blood and hematopoietic diseases, immune system diseases		
Hypereosinophilic syndrome	0	1
Thrombocytopenia	0	1
Sarcoidosis	0	1
Endocrine, nutritional, and metabolic diseases		
Hypothyroidism	1	1
Graves' disease	1	2
Type 2 diabetes	2	3
Diabetes (unspecified)	3	3
Hypopituitarism	0	1
Obesity	2	3
Dyslipidemia	2	6
Hyperuricemia	2	0

Familial Mediterranean fever	0	1
Mental and behavioral disorders		
Bipolar disorder	0	1
Depressive disorder	1	7
Dysthymia	0	1
Panic disorder	0	1
Anxiety disorder	0	1
Attention deficit hyperactivity disorder	0	1
Neurological diseases		
Migraine	0	2
Insomnia	0	1
Sleep apnea syndrome	8	6
Sleep disorder	1	0
Carpal tunnel syndrome	1	0
Hemiplegia	0	1
Ophthalmologic diseases		
Dry eye	0	1
Allergic conjunctivitis	0	1
Cataract	4	1
Macular degeneration/epiretinal membrane	3	2
Glaucoma	3	3
Hemiamaurosis	0	1
Cardiovascular diseases		
Hypertension	13	8

Angina pectoris	1	1
Löffler's endocarditis	0	2
Cardiomyopathy	0	1
Atrial fibrillation	1	0
Premature ventricular contraction	1	0
Arrhythmia	4	0
Arteriosclerosis	1	0
Respiratory system diseases		
Pollinosis	8	22
Allergic rhinitis	3	7
Eosinophilic sinusitis	0	2
Sinusitis	0	2
Nasal polyps	0	1
Chronic obstructive pulmonary disease	3	0
Eosinophilic pneumonia	2	1
Pleural plaque	1	0
Digestive system diseases		
Periodontal disease	3	1
Reflux esophagitis	4	1
Gastroesophageal reflux disease	2	1
Duodenal ulcer	1	0
Gastritis/chronic gastritis	1	3
Ulcerative colitis	0	1
Constipation	2	2

Colorectal polyp	3	1
Intestinal adhesion	0	1
Gallbladder polyp	0	1
Skin and subcutaneous tissue diseases		
Atopic dermatitis	5	3
Pruritus	0	1
Urticaria	0	2
Alopecia areata	1	0
Acne	0	1
Musculoskeletal and connective tissue diseases		
Rheumatoid arthritis	0	2
Gout	1	0
Osteoarthritis	0	5
Arthralgia	1	1
Eosinophilic granulomatosis with polyangiitis	1	2
Anti-neutrophil cytoplasmic antibody-associated vasculitis	0	1
Spinal canal stenosis	1	0
Sciatica	0	1
Low back pain	1	2
Osteoporosis	0	2
Renal and urogenital diseases		
Benign prostatic hyperplasia	2	0
Endometriosis	0	1
Endometrial hyperplasia	0	1

Premenstrual syndrome	0	1
Congenital malformations and chromosomal abnormalities		
Congenital cataract	0	1
Developmental dysplasia of the hip	0	1
Effects of injuries and other external causes		
Cerebral contusion	0	1
Food allergy	0	4
Latex allergy	0	1
Others		
Headache	0	1
Malaise/fatigue	2	3
Underweight	0	1
Sensitivity to cold	1	2
Long COVID	0	1
Artificial joint	0	2

Abbreviations: BA, bronchial asthma; COVID, coronavirus disease

Table S3. List of diagnosed diseases confirmed at the time of stool sample collection for the gut microbiota testing service

1. Heart disease
2. Kidney disease
3. Liver disease
4. Stomach disease
5. Intestinal disease
6. Colon cancer
7. Colon polyps
8. Cancers other than colon cancer
9. Type 1 diabetes
10. Type 2 diabetes
11. Hypertension
12. Dyslipidemia
13. Obesity
14. Underweight
15. Bone and joint diseases
16. Lower back pain, joint pain
17. Asthma
18. Allergic rhinitis
19. Food, drug, and metal allergies
20. Atopic dermatitis
21. Autoimmune diseases, immune disorders
22. Depression

23. Stress-related disorders other than depression

24. Eye diseases

25. Oral diseases

26. Ear disease

27. Cold

28. Injuries

29. Other diseases

Table S4. Distribution of participants by obesity status in the BA and control groups, stratified by sex

BMI	Male individuals		Female individuals	
	BA group (*n* = 48)	Control group (*n* = 90)	BA group (*n* = 60)	Control group (*n* = 120)
<25	27	66	41	112
≥25	21	24	19	8

Abbreviations: BA, bronchial asthma; BMI, body mass index
